# Characterization of a valproic acid-sensitive mutant allele of the Golgi GDP-mannose transmembrane transporter Vrg4 in *Schizosaccharomyces pombe*

**DOI:** 10.17912/micropub.biology.001287

**Published:** 2024-08-23

**Authors:** Teruaki Takasaki, Minami Yamada, Haruka Ikeda, Yue Fang, Reiko Sugiura

**Affiliations:** 1 Department of Pharmaceutical Sciences, Faculty of Pharmacy, Kindai University, Osaka, Japan; 2 Department of Microbial and Biochemical Pharmacy, School of Pharmacy, China Medical University, Shenyang, Liaoning, China

## Abstract

Valproic acid (VPA) is a widely used drug for epilepsy. However, precise molecular mechanisms relevant to VPA's side effects remain elusive. This study identifies a VPA-sensitive mutant strain ( *vas21* ) in fission yeast with a missense mutation (T256I) in the nucleotide sugar-binding motif of the GDP-mannose transporter Vrg4 . This mutation impairs protein glycosylation, as evidenced by altered acid phosphatase mobility. We also found that Vrg4 overexpression deteriorates cell growth. Our results highlight the role of Vrg4 in glycosylation and implicate impaired glycosylation as a potential mechanism underlying VPA sensitivity. The new allele of *vrg4* will be useful in glycobiology and pharmacology.

**Figure 1. Characterization of valproic acid sensitive mutant vas21:**
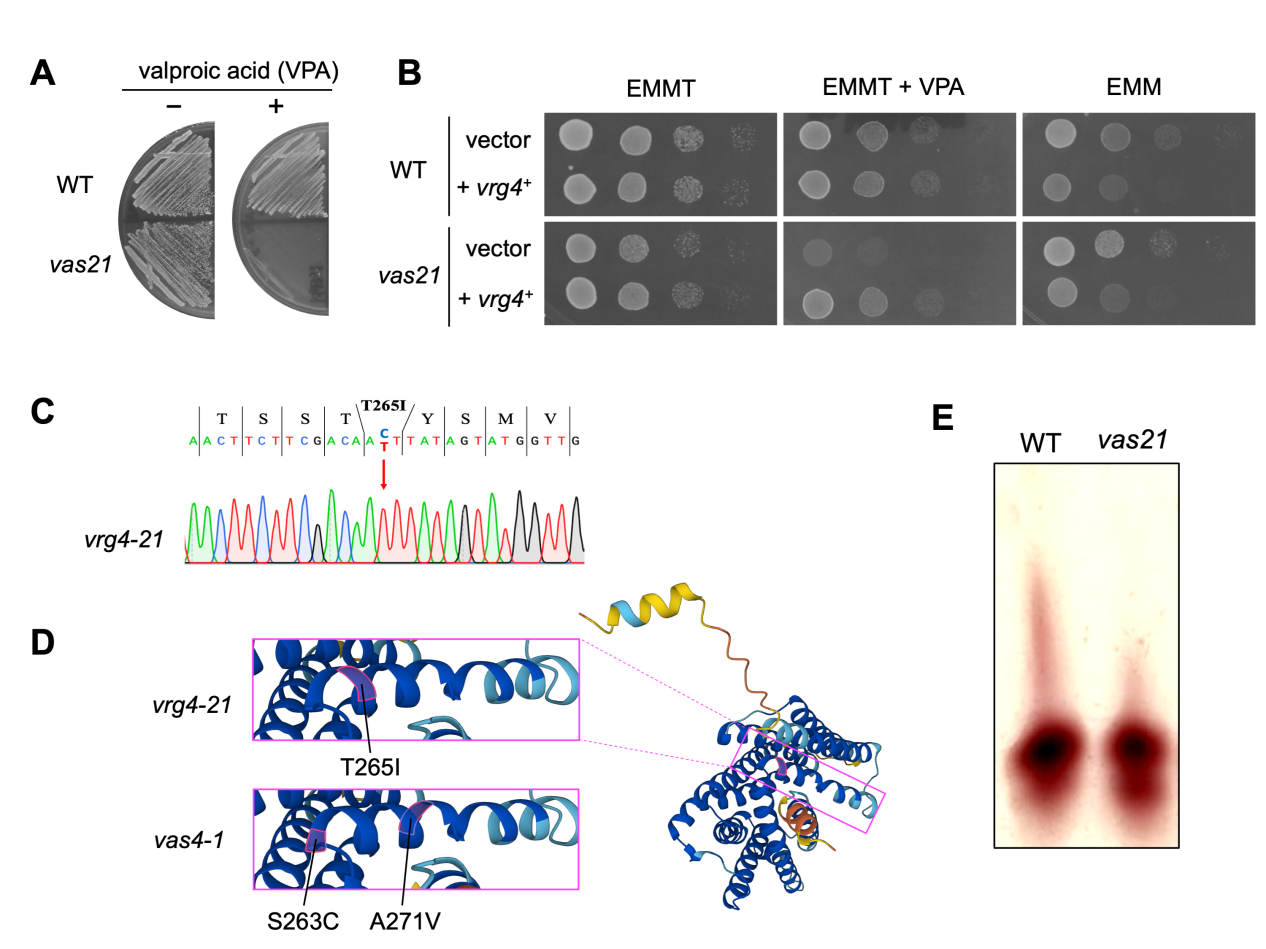
**A:** Wild-type (WT) and *vas21* mutant strains transformed with pREP1-GFP vector were streaked onto the Edinburgh Minimal Media (EMM) plus thiamine (EMMT) plates with or without 6 mM VPA and incubated for 3 days at 27°C. **B:** WT and *vas21* strains were transformed with the control vector (pREP1-GFP) or the vector containing *vrg4* ^+^ and spotted as indicated on the EMM or EMMT plates with or without 6 mM VPA in serial 10-fold dilutions. The plates were incubated for 3 days at 27 °C. **C:** DNA sequencing revealed a missense mutation in the *vrg4-21* allele. The arrow indicates the single nucleotide change from C to T in the 265 ^th^ codon. **D:** Positional relationship of the *vrg4-21* and *vas4-1* mutation sites. The images of the predicted 3D structure were obtained from PomBase (www.pombase.org) (Jumper et al. 2021; Varadi et al. 2024; Rutherford et al. 2024). **E:** Acid phosphatase glycosylation in WT and *vas21* mutant cells. Cell lysates were separated by 6% native polyacrylamide gel electrophoresis and acid phosphatase was stained with Fast Blue B salt and 1-naphthyl phosphate.

## Description

Valproic acid (VPA, 2-n-propyl pentanoic acid) is a short-chain fatty acid that is widely prescribed as a medication for the treatment of epilepsy, bipolar disorders, and migraine prophylaxis (Linde et al. 2013; Romoli et al. 2019) . It was first approved for use as an antiepileptic agent in France in 1967 and is now approved in more than one hundred countries (Bhushan 2003) . Although VPA activity as an anti-convulsant is considered to be mediated by a rise in glutamatergic and γ-aminobutyric acid in the brain, recent progress unraveled novel pharmacological activities associated with VPA, including inhibition of histone deacetylases (HDACs) or blockade of voltage-gated ion channels. Thus, VPA has attracted increasing attention as a versatile drug with multifaceted mechanisms of action promising for various diseases, including certain types of cancers, mellitus, kidney disorders, neurodegenerative diseases, cardiovascular disorders, and muscular dystrophy (Singh et al. 2021) .

VPA can induce various side effects, including vomiting, heartburn, nausea, weight gain, dermatological side effects dosage-related tremors, and neurological side effects including ataxia, sleepiness, and irritability. It can also induce some serious disorders, such as thrombocytopenia, hyperammonemia, Parkinsonism, and birth defects (DiLiberti et al. 1984; Nau et al. 1991; Ibadova 2017; Baddour et al. 2018; Muralidharan et al. 2020) . Predicting a patient's response to VPA remains difficult, in part because the relevance between side effects and genetic predisposition is unclear.

To gain insight into the molecular basis that could influence VPA's efficacy and side effects, we have previously established a genetic screening for V alproic A cid S ensitive ( *vas* ) strains in fission yeast that had been mutagenized with nitrosoguanidine (Zhang et al. 2000) . This screening has successfully identified several mutations that were mapped to the *vps45* , *aps1* , and *vrg4* loci (Miyatake et al. 2007; Ma et al. 2009; Qiao et al. 2021) . Vrg4 is a GDP-mannose transporter localized to the Golgi, which is crucial for glycoprotein modification (Bredeston et al. 2016) . One of our *vas* strains *vas4-1* has been shown to harbor double missense mutations (S263 and A271) in the nucleotide sugar-binding motif of Vrg4 . Furthermore, although the two mutation sites had little effect on the overall structure of Vrg4 , they impaired the glycosylation of proteins, including the cell surface glycoprotein acid phosphatase (Qiao et al. 2021) . In this study, we identified another allele of *vrg4* by analyzing the previously uncharacterized *vas* strain ( *vas* 21).

As shown in Figure 1A, the *vas21* mutant strain grew similarly (only slightly slower) to the wild-type cells under normal culture conditions. However, the *vas21* mutant cells exhibited a significant reduction in growth in the medium containing 6 mM VPA, a concentration that did not influence the growth of WT cells. To identify the mutated gene, we screened a fission yeast genomic library and cloned the *vrg4* ^+^ gene that complements the VPA sensitivity of *vas21* mutant cells. The result was confirmed by subcloning the *vrg4* ^+^ gene into the thiamine-regulatable expression vector pREP1, which induces a wide dynamic range of expression, with low expression in the presence of thiamine and high expression in the absence of thiamine (Maundrell 1993; Moreno et al. 2000) . The growth defect of *vas21* on the VPA-containing medium was fully recovered by transformation with the vector containing *vrg4 ^+^* ( Figure 1B, middle panels). Therefore, considering the involvement of *vrg4 ^+^* in the VPA sensitivity of *vas21,* we designated *vas21* as *vrg4-21.* As an unexpected finding, high-level induction of Vrg4 deteriorated cell growth in both WT and *vas21* cells despite the absence of VPA ( Figure 1B, right panels). Therefore, overexpression of Vrg4 may induce a toxic effect on cell growth.

To identify the mutation site, the *vrg4* locus of the *vrg4-21* allele was isolated by PCR amplification and subjected to Sanger sequencing. A single nucleotide change was identified at the 256th codon, which causes a substitution from the hydrophilic Thr residue to hydrophobic Ile in the nucleotide sugar-binding motif ( Figure 1C ). Notably, the mutated residue in *vrg4-21* is located between the two mutated residues found in *vas4-1* ( Figure 1D ).

Given that Vrg4 is a putative Golgi-located GDP-mannose transporter, we examined the impact of the *vrg4-21* mutation on glycosylation. For this purpose, we analyzed the mobility of acid phosphatase—a well-documented substrate for *N* -linked glycosylation (Schwaninger et al. 1990) and a well-established marker for investigating impairments in glycosylation status induced by mutations (Huang and Snider 1995; Ohashi et al. 2020; Tanaka et al. 2021) —using native gel electrophoresis. We found that acid phosphatase isolated from *vas21* mutant cells migrated signiﬁcantly faster than that from WT cells ( Figure 1E ), suggesting the impaired protein glycosylation in *vas21* mutant.

Collectively, our data are consistent with the previous finding that the amino acid sequences in the nucleotide sugar-binding motif of Vrg4 are important for conducting proper glycosylation. However, it is still unclear why the malfunction of Vrg4 affects the sensitivity to VPA. In budding yeast, Vrg4 p is essential for cell wall integrity (CWI) and normal Golgi function, and the null mutant is lethal (Poster and Dean 1996; Dean et al. 1997) . Fission yeast ∆ *vrg4* mutant cells also display severe growth defects with impaired cell wall synthesis, morphological aberrations, and agglutination tendencies (Bredeston et al. 2016) . Considering that the null mutants are more severe than the missense alleles, VPA may exert the toxic effect by further attenuating the reduced function of Vrg4 , for example, by suppressing the expression or localization of Vrg4 to the Golgi. Alternatively, the impaired glycosylation may cause sensitivity to VPA. In eukaryotes, glycosylation serves a crucial role in cell physiology and impacts numerous processes, including quality control during protein folding, protein trafficking, cell recognition, developmental signaling, and immune system function (Reily et al. 2019) . Further studies will need to clarify the cell physiology that affects VPA sensitivity.

## Methods

**Yeast strains and medium.** *S. pombe* strains used in this study are listed in the Reagents section. Strains were grown in Edinburgh minimal medium (EMM) as described previously (Sabatinos and Forsburg 2010) . Unless otherwise stated, media were supplemented with 5 µM thiamine. Valproic acid was purchased from Sigma (St. Louis). Spot assays were performed three times with reproducible results.

**Isolation of the *vas21* mutant and identification of the mutation site.** The *vas21* mutant was identified through a screening of cells that had been mutagenized with nitrosoguanidine as previously described (Zhang et al. 2000) . *vrg4 ^+^* gene was cloned by complementation test using an *S. pombe* genomic DNA library constructed by the method described previously (Beach et al. 1982) . *vrg4-21* allele was amplified by PCR with oligonucleotides (CGGGATCCATGGATAATCATATGCTAAACC and GACTTTGACAGACTATCGCG) and the PCR products were analyzed by Sanger DNA sequencing by Macrogen Japan Corp. (Tokyo, Japan).

**Acid phosphatase staining** Acid phosphatase from ﬁssion yeast was separated by a non-denaturing polyacrylamide gel electrophoresis (PAGE) and stained as described in (Schweingruber et al. 1986) , with some modiﬁcations. Briefly, cells were grown in 20 ml of EMM medium to mid-log phase and then replaced with phosphate-free EMM followed by 7 h incubation at 27˚C to induce the production of acid phosphatase. Cells were then collected by centrifugation, washed once with 62.5 mM Tris-HCl (pH 6.8), and suspended in ice-cold lysis buffer (62.5 mM Tris-HCl, 1 mM EDTA, 2 mM phenylmethylsulfonyl ﬂuoride, 0.1 mM dithiothreitol and 10% glycerol, pH 6.8) and homogenized with glass beads using Multi-beads Shocker (Yasui Kikai, Osaka, Japan). The lysates were cleared by centrifugation at 15,000 rpm for 10 min. The supernatant was mixed with a one-third volume of 0.01% bromophenol blue, 15% glycerol and 62.5 mm Tris-HCl (pH 6.8). Samples were separated by native-PAGE (6% polyacrylamide) and the gels were immersed in 100 mM sodium acetate (pH 4.0) for 15 min and then stained with Fast Blue B salt and 1-naphthyl phosphate as described in (Miyatake et al. 2007) . The mobility assays for acid phosphatase were performed four times with reproducible results.

## Reagents

**Table:** 

**Strains**	**Genotype**	**Reference**
HM123	* h ^-^ leu1-32 *	Lab stock
KP1331	* h ^-^ leu1-32 vrg4-21 *	Lab stock
		
**Plasmids**	**Description**	**Reference**
pKB2728	pREP1-GFP	Lab stock
pKB4886	pREP1-GFP- *vrg4 ^+^*	This study
